# A Rare Case of Idiopathic Plastic Bronchitis

**DOI:** 10.1155/2017/4789751

**Published:** 2017-10-02

**Authors:** Mohammed Raoufi, Leila Achachi, Fatima Zahra Mrabet, Laila Herrak, Mustapha El Ftouh, Najoua Bourhroum, Nezha Ouazzani Taibi

**Affiliations:** ^1^Avicenne University Hospital, Pulmonary Unit, University of Rabat, Rabat, Morocco; ^2^Avicenne University Hospital, Anatomopathology Unit, University of Rabat, Rabat, Morocco; ^3^Avicenne University Hospital, Radiology Unit, University of Rabat, Rabat, Morocco

## Abstract

Plastic bronchitis is a rare disorder characterized by formation of large, branching bronchial casts, which are often expectorated. We present an interesting case of a 35-year-old woman who presented for evaluation of a chronic cough productive of voluminous secretions. Clinical and radiological examination confirmed a total left lung atelectasis without any pathological mediastinal node. Flexible bronchoscopy demonstrated tenacious, thick, and sticky whitish secretions blocking the left stem bronchus. This material was extracted, and inspection demonstrated a bronchial cast, whose pathological analysis revealed necrotic epithelial cells, some eosinophils, and Charcot-Leyden crystals. Two days after bronchoscopy, the patient rejected more bronchial casts, and dyspnea improved. Control of chest x-ray revealed complete left lung aeration and the diagnosis of idiopathic plastic bronchitis was obtained. This article shows the interest in clinical practice to evoke the diagnosis of plastic bronchitis in front of a productive chronic cough. Our case illustrates a rare clinical presentation represented by an atelectasis of an entire lung.

## 1. Introduction

Plastic bronchitis is a rare disease characterised by the formation of large gelatinous or rigid airway cast. Plastic bronchitis is probably more common than reported. This speculation is based on the observation that many clinicians are unfamiliar with the disease and may fail to recognize milder forms of the syndrome. We present a case of idiopathic plastic bronchitis occurring in an adult.

## 2. Observation

A 35-year-old woman with no history of lung disease or occupational exposures and no active or passive cigarette smoking.

She had presented for evaluation of a chronic cough productive of voluminous secretions.

Over the previous 3 months, she had experienced episodic wheezing and dyspnea. During these episodes she often expectorated thick secretions which she described as resembling squid. Admitted to the pulmonary department she had no other respiratory or expiratory symptoms.

**Figure 1:**
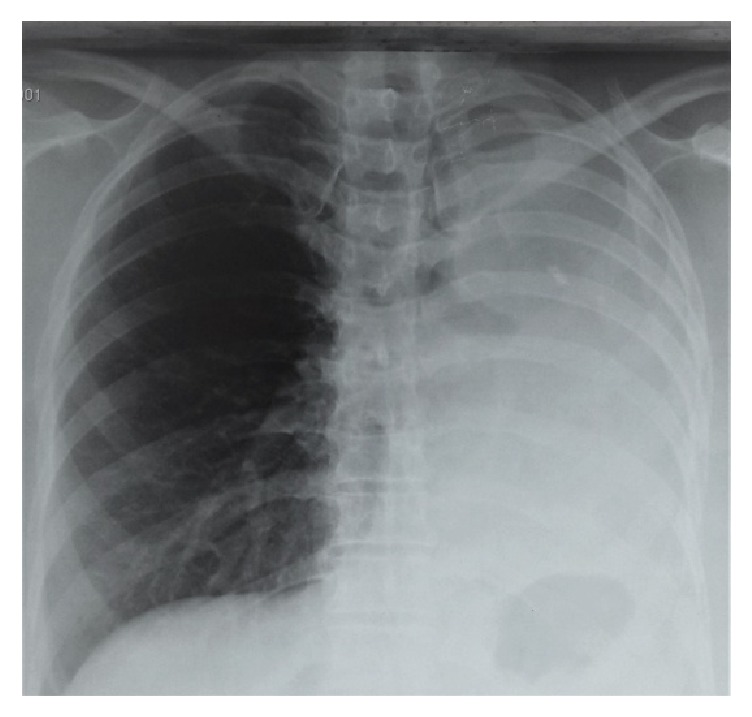
Chest X-ray showed a large opacity of the entire left hemithorax with attraction of the mediastinum element towards the opacity.

In clinical examination, heart rate was 96 bpm and polypnea was at 26 cpm, with condensation syndrome of all the left hemithorax. The chest-X ray showed a large opacity of the entire left hemithorax with attraction of the mediastinum element towards the opacity (Figure 1).

**Figure 2:**
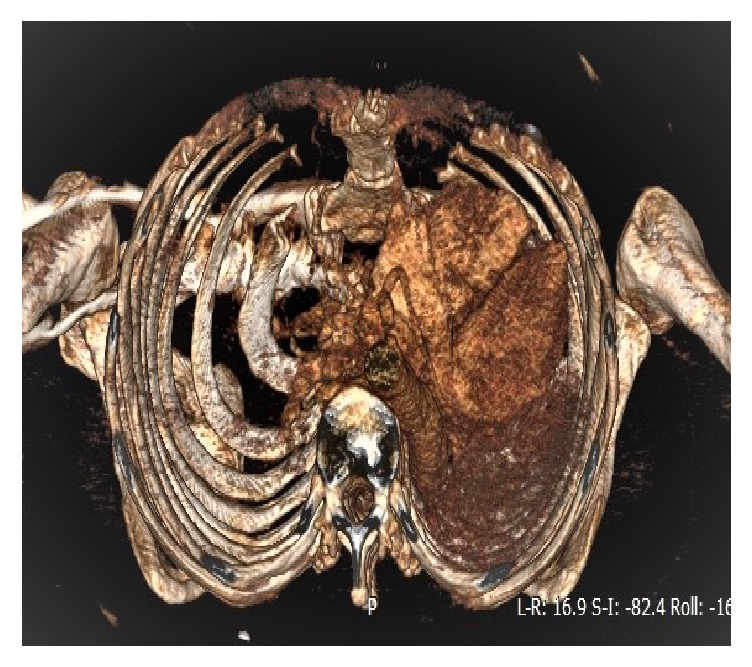
3D CT scan in axial tomography of the chest confirmed a total left lung atelectasis.

Computerised axial tomography of the chest confirmed a total left lung atelectasis without any pathological mediastinal node, oriented towards an endobronchial obstacle (Figure 2).

The x-pert MTB/RIF detects no DNA sequences specific for mycobacterium tuberculosis in specimen sputum. Flexible bronchoscopy demonstrated tenacious, thick, and sticky whitish secretions blocking the left stem bronchus.

**Figure 3:**
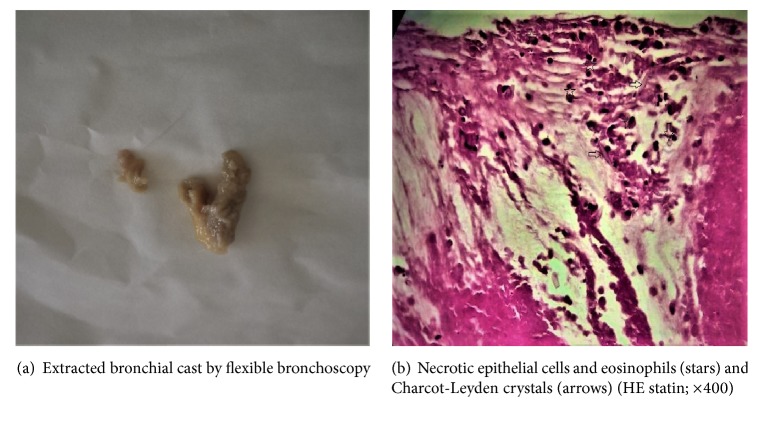


**Figure 4:**
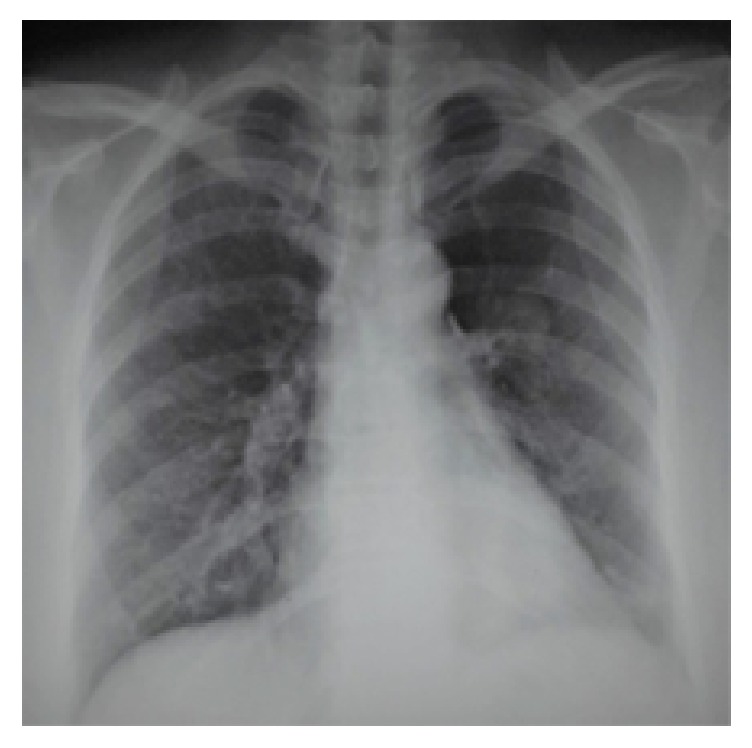
Control of chest-x-ray after cast rejection revealed complete left lung aeration.

This materiel was extracted, and inspection demonstrated a bronchial cast, whose pathological analysis revealed necrotic epithelial cells, some eosinophils, and Charcot-Leyden crystals (Figures 3(a) and 3(b)). Two days after bronchoscopy, the patient rejected more bronchial casts, and dyspnea improved. Control of chest-x-ray revealed complete left lung aeration (Figure 4).

The diagnosis of idiopathic plastic bronchitis was retained.

Despite several therapies with antimicrobials, glucocorticoids, and inhaled hypertonic saline, the patient continued to have extensive cast production over the next year.

She underwent repeat flexible bronchoscopy and small casts are rejected continually; she is always followed-up in the pulmonary department.

## 3. Discussion

Plastic bronchitis (PB) is an uncommon pulmonary disease characterized by production of cohesive and branching casts filling the airways. PB has been previously identified by fibrinous bronchitis, bronchitis pseudomembranosa, and Hoffmann bronchitis and is now uniformly termed plastic bronchitis. The prevalence of PB is unknown. Many patients remain undiagnosed. This disorder can occur at any age, but most publications interest children population [1].

The formation of bronchial casts in plastic bronchitis is extremely variable; it goes from small fragmented bronchial casts—as our patient's case—to enormous casts filling the airways of an entire lung.

The bronchial casts are differentiated to inflammatory and acellular cast. Type 1 is often associated with underlying diseases. It is characterized by an acute presentation. The second type has often a chronic history of clinical symptoms [2].

Several systemic illnesses, cardiac diseases, pulmonary diseases, and disorders of lymphatic drainage have been associated with plastic bronchitis. In our case, no pathology or associated disease was found.

The clinical presentation has productive cough, dyspnea, pleuritic chest pain, fever, and wheezing. Radiographic evaluation reveals the site of the bronchial cast impaction, demonstrating atelectasis or infiltrates. Hyperinflation is often evident on the contralateral side. The CT scan allows visualization of impacted casts within the major airways [3].

The diagnosis of plastic bronchitis is confirmed by recovery of casts that have been coughed up or visualized during a bronchoscopy. There is no specific cytological, pathologic, or laboratory test that is diagnostic for casts due to lymphatic PB.

An etiological check-up and an exhaustive search for diseases which may give bronchial secretions should be done before retaining this diagnosis. And lung cancer must always be among the diagnoses to be evoked.

Therapy for PB is often focused on removal or facilitated expectoration of the casts and treatment of underlying pathology. Because PB is an uncommon condition, most reports of effective therapy are based on subjective criteria detailed in case reports or small case series.

If the clinical presentation is acute, such as dyspnea and hypoxemia caused by atelectasis of an entire lung, management should be in intensive care, and flexible bronchoscopy after stabilization of the patient is necessary.

Casts must be removed, mechanically by bronchoscopy or physical therapy. High-frequency chest wall oscillation can also be used to vibrate the chest wall at a high frequency to try to loosen and thin the casts [4, 5].

We used in our case nebulization with hypertonic salty serum and respiratory kinesitherapy to fluidize and facilitate the drainage of the casts.

It has been reported that inhalation of TPA and heparin can improve patients with BP, but there are many adverse effects limiting their use [6].

The prognosis of plastic bronchitis is generally favorable if the disease is properly treated at the beginning and if the research of associated diseases is negative. By contrast, PB secondary to cyanotic congenital heart disease is often associated with a rather grim prognosis with respiratory failure secondary to central airway obstruction as a common mechanism of death.

## Abbreviations

MTB/RIF: Mycobacterium tuberculosis
PB: Plastic bronchitis.
